# Comparison of student performance by assessment through Objective Structured Practical Examination versus the Conventional Method for second year MBBS students in Microbiology

**DOI:** 10.30476/jamp.2020.86029.1210

**Published:** 2020-07

**Authors:** MONIKA RAJANI, BABAJI GHEWADE

**Affiliations:** 1 Department of Microbiology, Career Institute of Medical Sciences and Hospital, IIM road, Lucknow, Uttar Pradesh, India; 2 Department of Respiratory Medicine, J N Medical College, Wardha Sciences and Hospital, Wardha, Maharashtra, India

**Keywords:** Teaching, Medical education, Assessment, Microbiology

## Abstract

**Introduction:**

Objective Structured Practical Examination (OSPE) is a comprehensive tool for assessment. We wanted to improve our assessment methods and make it a more competence-based evaluation. Thus this study was designed to compare the effectiveness of Objective Structured Practical Examination with that of Conventional Practical Examination.

**Methods:**

This interventional study was carried out in Department of Microbiology atCIMSH, Lucknow over six months from October 2019 till March 2020. One hundredsecond year MBBS students were enrolled. The students were divided into two groups offifty for the conventional examination group (controls) and the OSPE group.On the first day, the cases appeared for OSPE while the controls for conventional examination.On the second day, the groups were crossed over. The students appearing for OSPEwere assessed by their scores at different stations. Feedback forms with a prestructured questionnaire were given to the students and the examiners after OSPE on both days to record their perceptions. Finally, the students’ scores were tabulated and comparedstatistically. Microsoft Excel and SPSS were used for data analysis. The data was presented as percentages, mean and standard deviation. Student t test was used and the significance was checked, using p value <0.05.

**Results:**

Overall in OSPE, the students scored higher and the result was statisticallysignificant. The proportion of students in higher marks range was more for OSPE thanthat for the conventional method. The difference was statistically significant (p<0.001).Feedback taken from examiners as well as from the students in the form of astructured questionnaire to analyze their perceptions was very encouraging.

**Conclusions:**

OSPE is a comprehensive assessment modality for practical evaluationof MBBS students. OSPE proved to be an effective tool that improved the students’scores in microbiology.

## Introduction

Assessment is an integral component of competency-based medical education. Assessment drives learning but to foster active learning, assessment needs to be informative ( 1 ). With revised medical curriculum, we have moved from a standard of pen-and-paper tests of knowledge toward a more complex system of evaluation ( 2 ).

New assessment tools have been designed to test not only knowledge but also practical, procedural and communication skills with meaningful feedback to the students and Objective Structured Practical Examination (OSPE) is a comprehensive assessment tool for this purpose.

The term OSPE was derived from Objective Structured Clinical examination in 1975 which was later extended to practical examination and was modified by Harden and Gleeson ( 3 ). It can be used as an evaluation as well as teaching tool.

The OSPE assesses practical competencies in an objective and structured manner with direct observation of the students’ performance during planned clinical test stations. OSPE is a comprehensive tool to test psychomotor skills at procedural station, knowledge at question station, and also ability to observe, analyze and interpret.

The examination can be structured to achieve the desired matrix of different elements being assessed, with each element receiving the desired weightage. A traditional practical examination in Microbiology focuses on the “knows” and “knows how” aspects and is inadequate in evaluating the overall performance of the students. OSPE is based on global performance rather than the candidate’s individual competency. Other problems encountered are patient and examiner variability, which significantly affects the student’s scores ( 4 ).

On the other hand, OSPE focuses on the “shows how” aspect of Miller's pyramid of competence focusing on the assessment of performance of specific skills in a controlled setting. OSPE promotes improved reliability, validity and unbiased method of assessment as all the candidates are represented to the same task ( 5 ).

Our institute follows conventional method of practical examination. We wanted to improve our assessment methods and make it more competence-based, thus overcoming the limitations associated with conventional practical examinations. Thus this study was designed to compare the effectiveness of Objective Structured Practical Examination with the Conventional Practical Examination.

## Methods

This interventional study was carried out in the Department of Microbiology at Career Institute of Medical Sciences and Hospital, Lucknow for a period of six months from October 2019 to March 2020. Permission was taken by Institutional Ethics committee before commencing the study.

One hundred students of second year MBBS fifth semester batch 2017-18 were enrolled. The second year MBBS batch has hundred students. All of the students participated in the study. The students were randomized into two groups by lottery method. They were sensitized about OSPE by lectures and demonstrations. The faculty, residents and tutors were also sensitized to OSPE. OSPE was structured by trained faculties in Microbiology. Ten stations were structured out of which six stations were procedural stations and four were question stations. Each station was allotted four marks. Each task/question was broken into subtasks /sub questions and the marks were divided accordingly. On procedural stations, the observer scored each subtask as the pre-validated checklist. All scores were entered into the excel sheet and evaluated.

The time allotted to each station was four minutes. The maximum score for both examinations was forty. The preparation of question cards and the model answers for the question stations and checklist validation for procedural stations were done by senior faculty members.

The examination was given on two consecutive days in January, 2020.

The students were divided randomly into two equal groups, fifty students in the conventional examination group (controls) and fifty in the OSPE group.

- DAY 1: On the first day, roll numbers 51 to 100 (N51-N100) appeared for OSPE as cases while roll numbers 1-50 (N1-N50) appeared as controls for the conventional examination.

- DAY 2: The study and the control group crossed over on day two of the examination. This was done to eliminate any bias and give strength to the study.

The exercises selected for conventional examination and OSPE on the two days were different but of the same difficulty level to avoid any bias. Pre-validated checklists were used at procedural stations. The students appearing for OSPE were assessed by their scores at different stations. The students who appeared for the conventional examination were assessed by scores in viva voce at the end of examination. Feedback forms with a pre-structured questionnaire were given to the students after OSPE on both days to record their perceptions. Feedback was also taken from examiners through a separate questionnaire. Finally, the students’ scores in the conventional examination and OSPE were tabulated and compared statistically. Microsoft Excel and SPSS were used for data analysis. The data was presented as percentages, mean and standard deviation. Student t-test was used and the significance was checked, using p value <0.05. Responses in the feedback questionnaire were also evaluated.

## Results

Overall, the mean score obtained by the students in conventional method was 18.26±4.33 while in OSPE method it was 23.36±6.79 and so a difference of 5.11 was seen (Table 1), which was statistically significant (p<0.001). So overall in OSPE, the students gained significantly higher scores.

**Table 1:** Overall comparison of Mean Marks Obtained through the two methods

Overall	Mean±SD	Mean Diff.	t	p
Conventional	18.26±4.33	5.11	16.51	<0.001
OSPE	23.36±6.79			

**Figure 1:**
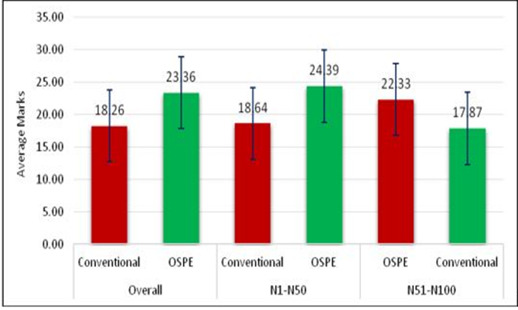
Comparison of Mean Marks Obtained overall by the two groups

Both groups gained higher marks in OSPE and the difference was statistically significant as shown in Table 2 and Figure 1.

**Table 2:** Comparison of Mean Marks of the two groups in conventional exam and OSPE

(N1-N50) Conventional followed by OSPE	Mean± SD	Mean change	t	p	N51-N100 OSPE followed by Conventional	Mean± SD	Mean change	t	p
Conventional	18.64±4.34	5.75	13.86	<0.001	OSPE	22.33±6.84	4.46	10.04	<0.001
OSPE	24.39±6.66				Conventional	17.87±4.33			

**Figure 2:**
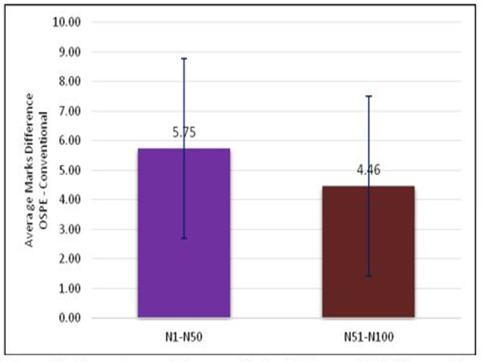
Comparison of Average Marks Difference (OSPE-conventional) between the two groups

Both groups had a higher score in the OSPE than that in the conventional examination and the mean score difference was statistically significant (p=0.036) (Figure 2).

**Figure 3:**
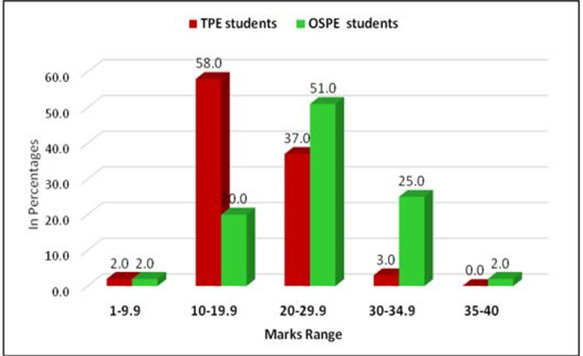
Comparison of proportion of Marks gained by the two Methods

The proportion of students in higher marks range was more for OSPE than for conventional method i.e. 25% as compared to 3% in the marks range 30-34.9 and 2% as compared to 0% in 35-40 marks range. The proportion difference was highly significant (p<0.001) as depicted in Figure 3.

**Figure 4:**
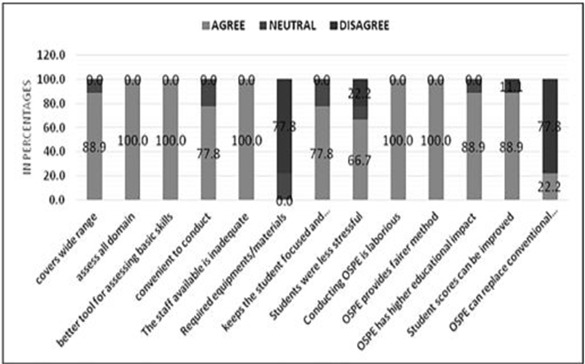
Faculty and examiner feedback

Faculty and examiner feedback were taken in the form of a structured questionnaire, their responses being shown in Figure 4.

**Figure 5:**
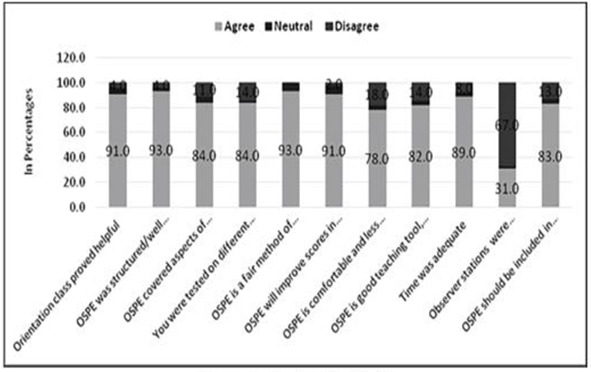
Student feedback

The student feedback was taken in the form of a structured questionnaire from the OSPE group on both days, the responses being shown in Figure 5.

## Discussion

The current study was undertaken to introduce and compare the effectiveness of OSPE with the conventional method in the department of microbiology on 100 second year MBBS students.

Overall, the difference in average marks obtained by the students in the OSPE method and the conventional method was statistically significant (p<0.001). Thus the students gained higher marks in OSPE. The proportion of students in higher marks range was more for OSPE than for the conventional method i.e. 25% as compared to 3% in the marks range, 30-34.9 and 2% as compared to 0% in the marks range, 35-40. The proportion difference was highly significant (p<0.001). Various authors have yielded similar results ( 2 , 6 , 7 ). Ashok et al. ( 6 ) compared the conventional method of assessment with OSPE and observed a significant improvement in the scores obtained by the students in OSPE (p <0.001). The studies done by Patil et al. ( 2 ) and Nigam R et al. ( 7 ) demonstrated similar findings.

In our study there was a definite improvement in the scores obtained in OSPE. The probable reason for the lower scores in the conventional examination was because of the assessment format where the students were assessed by viva voce to different faculties after the end of exercise and it was mainly dependent on memorization, recall, and also luck to some extent. Various limitations with this pattern like student variability, practical task variability and examiner variability significantly affect scoring.

Though OSPE was a new assessment format for our students, they still managed to score well because of the prior sensitization sessions, demonstrations and practice sessions. In OSPE the students faced the same set of targeted questions and exercises at different stations. The assessment was based on demonstration of individual competencies. The objectivity of the examination also improved the students’ scores and examiner variability was also reduced making it a more valid system of assessment. OSPE has been shown to have a better scope for being structured so that all the objectives of laboratory teaching can be tested.

On comparison of average marks difference between conventional and OSPE in the two groups, the N1-N50 group gained more marks in the OSPE method and the mean difference in marks was statistically significant. On the whole, the performance of NI-N50 group was better than the other group. The overall scores of the N51-N100 group were lower as compared to the N1-N50 group.

Feedback was taken from examiners as well as from the students in the form of a structured questionnaire to analyze their perceptions to OSPE, which was quite encouraging. Overall, it reflected the acceptability of the method among students and teachers. Since all the students were exposed to similar types of questions with the same difficulty level, they felt that the checklist system is a fair and unbiased method with lesser element of luck playing any part in assessment. Various studies by Patil et al. ( 2 ), Hasan et al. ( 4 ), Ananthakrishnan et al. ( 8 ), Abraham et al. ( 9 ) and Nayar et al. ( 10 ) reported the acceptability of OSPE ( 2 , 4 , 8 - 10 ).

This study was done at a private college and in most of the private colleges, financial support from the administration is limited. Similarly, the staff strength is also just minimum to fulfill the MCI norms. In such practical scenarios resources examiners, time, expenses and intensive manpower required for preparation of stations is challenging for the faculty.

Both the OSPE and conventional techniques independently test different abilities. The students’ attitude and communication skills cannot be assessed only by OSPE. Many aspects of microbiology practical skills like acid fast staining exercise is a lengthy procedure, which is difficult to adjust in a 4-6 min station. Secondly, culture exercise with problem-based exercises are important aspects to teach clinical microbiology and it can be better assessed by viva voce instead of separate stations. OSPE should be used in those microbiology exercises which involve practical skill (Psychomotor domain) e.g. Gram staining exercise, Stool examination, motility testing while conventional examination can be used for culture exercises with problem-based scenarios and interpretation of laboratory reports where concepts of understanding can be better judged with comprehensive viva voce.

## Conclusion

The author concludes that although new to our assessment system, OSPE turned out to be useful practical experience for MBBS students in our institute.

OSPE proved to be an effective tool that improved the student scores in microbiology and should definitely be incorporated in the assessment system. The attitude of the students and examiners toward OSPE was positive and encouraging.

It allows for uniform and reproducible level of assessment and constructive feedback to students for improvement. It may not only improve the quality of the students’ performance in the laboratory exercise but may prepare them for their clinical years so that good clinicians may be produced.

The use of OSPE as a formative tool will help in modifying teaching-learning strategies so that both teachers and students can derive maximum benefit.

Both the OSPE and Conventional techniques independently test different abilities. A combination of the conventional examination and OSPE would be a successful tool for assessment of students in Microbiology department.

In future, OSPE can also be used as a tool for testing multiple dimensions of post graduate students’ performance in our institute.

## References

[1] Namrata K, Rekha K, Shailesh K ( 2016). Comparison Of Objective Structured Practical Examination To Conventional Practical Examination. Natl J Integr Res Med.

[2] Patil H, Patil VC, Karande GS, Patil SR, Patil VC ( 2016). Acceptability, Feasibility and Feedback Analysis of Perception for Objective Structured Practical Examination As an Assessment Tool in Undergraduate in Competency Based Medical Education. JKIMSU.

[3] Harden RM, Stevenson M, Wilson DW ( 1975). Assessment of clinical competencies using objective structured clinical examination. Br Med J.

[4] Medical Council of India (2019). Assessment Module for Undergraduate Medical Education Training Program.

[5] Mokkapati A, Pavani G, Dass SM, Rao MS ( 2016). Objective structured practical examination as a formative assessment tool for II MBBS microbiology students. Int J Res Med Sci.

[6] Ashok NS, Prathab AG, Indumathi VA ( 2013). Objective Structured Practical Examination as a tool for evaluating competency in Gram staining. J Educational Res & Med Teach.

[7] Nigam R, Mahawar P ( 2011). Critical analysis of performance of MBBS students using OSPE & TDPE: A comparative study. Natl J Community Med.

[8] Ananthakrishnan N (1993). Objective structured clinical/practical examination (OSCE/OSPE). J Postgrad Med.

[9] Abraham RR, Raghavendra R, Surekha K, Asha K ( 2009). A trial of the objective structured practical examination in Physiology at Melaka Manipal Medical College, India. Adv Physiol Educ.

[10] NayarU, Malik SL, Bijlani RL ( 1986). Objective structured practical examination: a new concept in assessment of laboratory exercises in preclinical sciences. Med Educ.

